# Nomogram for individually predicting overall survival in rectal neuroendocrine tumours

**DOI:** 10.1186/s12885-020-07328-9

**Published:** 2020-09-09

**Authors:** Xingyu Feng, Gengzhou Wei, Wei Wang, Yu Zhang, Yujie Zeng, Minhu Chen, Ye Chen, Jie Chen, Zhiwei Zhou, Yong Li

**Affiliations:** 1 Department of General Surgery, Guangdong Provincial People’s Hospital, Guangdong Academy of Medical Sciences, The Second School of Clinical Medicine, Southern Medical University, No. 106, Zhongshan Er Road, Guangzhou, P.R. China; 2 Department of Emergency Medicine, Department of Emergency and Critical Care Medicine, Guangdong Provincial People’s Hospital, Guangdong Academy of Medical Sciences, Guangzhou, P.R. China; 3 Department of Gastric Surgery, Sun Yat-sen University Cancer Center, State Key Laboratory of Oncology in South China, Collaborative Innovation Center for Cancer Medicine, No. 651 Dongfeng Road East, Guangzhou, 510060 P.R. China; 4 Department of Gastroenterology, the First Affiliated Hospital of Sun Yat-sen University, No. 58, Zhongshan Er Road, Guangzhou, P.R. China; 5 Department of Gastrointestinal Surgery, Sun Yat-sen Memorial Hospital of Sun Yat-sen University, Guangzhou, P.R. China; 6 Department of Gastroenterology, Nanfang Hospital of Southern Medical University, Guangdong Provincial Key Laboratory of Gastroenterology, Guangzhou, P.R. China

**Keywords:** Rectal neoplasms, Neuroendocrine tumours, Nomogram, Overall survival

## Abstract

**Background:**

This study aimed to develop a nomogram that predicts the overall survival (OS) of rectal neuroendocrine tumours (NETs).

**Methods:**

We retrospectively analysed 310 patients with rectal neuroendocrine tumours in 5 hospitals in southern China. All of the patients were assigned to the training set. A multivariable analysis using Cox proportional hazards regression was performed using the training set, and a nomogram was constructed. It was validated on a dataset obtained from the Surveillance, Epidemiology, and End Result (SEER) database of America (*n* = 547).

**Results:**

In the training set, the nomogram exhibited improved discrimination power compared with the WHO grade guidelines (Herrell’s C-index, 0.872 vs 0.794; *p* < 0.001) and was also better than the seventh AJCC TNM classification (Herrell’s C-index, 0.872 vs 0.817; *p* < 0.001). In the SEER validation dataset, the discrimination was also excellent (C-index, 0.648 vs 0.583, *p* < 0.001 and 0.648 vs 0.603*, p = 0.016*, respectively, compared with G grade and TNM classification). Calibration of the nomogram predicted individual survival corresponding closely with the actual survival.

**Conclusions:**

We developed a nomogram predicting 1- and 3-year OS of patients with rectal neuroendocrine tumours. Validation revealed excellent discrimination and calibration, suggesting good clinical utility.

## Background

Neuroendocrine tumours (NETs) represent a relatively rare neoplastic tumor which are originating from neuroendocrine cells and peptidergic neurons. In recent years, the incidence of NETs has been increasing [1, 2]. The incidence of rectal neuroendocrine tumours is the highest within the gastrointestinal tract [3] and has significantly increased [1]. However, outcomes of patients with rectal NETs remain uncertain.

Currently, the most commonly used predictive systems for NETs are the AJCC and the European Neuroendocrine Tumour Society (ENETS) TNM staging systems or the WHO grade guidelines, which are based on the mitotic count and Ki67 proliferative index. These systems lack other clinicopathological features that can influence outcomes such as age, sex, and tumour size. Thus, our objective is to create a system that takes clinicopathological features into consideration, hoping it will provide a more accurate prognosis and have utility in clinical practice and medical decision making.

A nomogram is a pictorial representation of a complex mathematical formula [4]. Medical nomograms are good methods for predicting outcomes among patients with cancer [5]. Many take clinical variables such as tumour grade, tumour size, and patient age and build prognostic models that predict the risk of cancer recurrence or mortality for individuals. A nomogram is a graphical calculation instrument based on any type of function including logistic regression and Cox PHs regression models. When we build a nomogram, each variable is listed separately with a corresponding number of points assigned to a particular magnitude of the variable, and the cumulative point score for all of the variables is matched to a scale of outcomes.

In the past few years, Nomogram have been a well-established method for predicting prognostic factors of tumors [6–10]. To date, however, none have researched rectal NETs. With this study, we have designed a nomogram that focuses on rectal NETs. It was developed using the data from a relatively large cohort of patients who were treated in five hospitals in southern China. This nomogram can predict individual 1- and 3-year overall survival rates. It was validated with a dataset from SEER.

## Materials and methods

We retrospectively analysed the data of 442 patients with rectal NETs who were treated in 5 hospitals in southern China. However, 102 patients were lost to follow up before 3 years, and 30 patients had missing values; therefore, these 132 patients were excluded. Thus, a total of 310 patients were included in this study from Sun Yat-sen University Cancer Center (SYSUCC, *n* = 143), the First Affiliated Hospital of Sun Yat-Sen University (SYSUFH, *n* = 56), Guangdong General Hospital (GGH, *n* = 54), Nanfang Hospital of Southern Medical University (SMUNH, *n* = 44), and Sun Yet-san Memorial Hospital of Sun Yat-sen University (SYSUMH, *n* = 13) from November 1993 to December 2013.

The variables evaluated were age, sex, tumour sizes, surgery procedure, G grade, the depth of tumour invasion (T), the number of metastatic lymph nodes (N), distant metastases (M), and TNM stage. All patients were followed for at least 3 years. An endoscopy with rectal magnetic resonance imaging (MRI) or endoscopic ultrasonography (EUS) at 6 and 12 months were required during the first year of follow-up. After that, the patients were followed every 6 to 12 months. Abdominal and pelvic multiphasic computed tomography (CT) or MRI were required. Measurement of the biochemical marker chromogranin A was considered if the patients had clinical symptoms.

All 310 patients were assigned to the training set. A multivariable analysis using Cox PHs regression was performed using the training set, and the nomogram was constructed.

For the validation dataset, we collected data from the SEER database. We included data from 547 patients treated from 2005 to 2013 who were diagnosed with rectal NETs and had follow-up for at least 3 years.

## Methods

### Construction of the nomogram

We use the dataset from the 5 hospitals in China as the training set. Continuous variables such as tumour size were fitted to a smoothed restricted cubic splines [11].

The classification of categorical variables was determined by their clinical significance, and they had been divided before the construction of the nomogram. A univariate and multivariable Cox proportional hazards models were made to select the characteristics related to the survival time. By identifying characteristics predictive for overall survival in the multivariate Cox model, a nomogram was constructed to predict 1- and 3-year OS rates.

### Validation of the nomogram

Nomogram validation included two components by using the SEER external validation set. First, Discrimination was evaluated using a concordance index (C-index), which estimates the probability of concordance between predicted and observed responses. Harrell’s C-index, which is appropriate for censored data, was used to evaluate the discrimination [12]. The 95% confidence interval for Harrell’s C index can be obtained by adding and subtracting 1.96 × Se (Standard error) from the C index. The second component was calibration which was.

performed by grouping all patients according to the predicted quartile nomogram and then comparing the mean value of the group with the corresponding actual survival OS (calculated by kaplan-Meier method). All analyses were performed using SPSS version 20 (IBM, Armonk, NY, USA) and R version 2.13.2 (http://www.r-project.org) via the design and survival packages. A *P*-value of < 0.05 was considered significant.

## Results

### Clinicopathologic characteristics of the patients

A total of 310 patients in China and 547 patients from the SEER databases with rectal NETs were included in this study. All patients were followed for at least 3 years. The longest follow-up time was 224 months. The median survival time was 44 months. A total of 35 and 146 patients died in the training and validation datasets, respectively. The clinicopathologic characteristics of the patients in the training dataset and validation dataset are listed in Table 1.

**Table 1:** The clinicopathologic characteristics of the training and validation sets

Variable	Training Set (***n*** = 310)		Validation Set (***n*** = 547)	
	No. of patients	%	No. of patients	%
**Median age (years)**	49.1 ± 13.6		58.7 ± 13.1	
**Sex**				
Male	195	62.9	276	50.5
Female	115	37.1	271	49.5
**Surgical treatment**				
Endoscopic resection	139	44.8	–	–
Transanal excision	50	16.1	–	–
Radical resection	89	28.7	–	–
No surgical treatment	33	10.6	–	–
**Tumour Size (cm)**				
**< 2**	250	80.6	286	52.3
**2–4**	28	9.0	98	17.9
**> 4**	32	10.3	163	29.8
**G classification**				
G1	235	75.8	226	41.3
G2	45	14.5	59	10.8
G3	30	9.7	262	47.9
**T Staging**				
T1	231	74.5	303	55.2
T2	38	12.3	81	14.8
T3	31	10.0	100	18.3
T4	10	3.2	64	11.7
**N Staging**				
N0	264	85.2	353	64.7
N1	46	14.8	194	35.3
**M Staging**				
M0	277	89.4	423	77.3
M1	33	10.6	124	22.7
**TNM Staging**				
**I**	220	71.0	267	48.8
**II**	31	10.0	48	8.8
**III**	26	8.4	108	19.7
**IV**	33	10.6	124	22.7

*TNM* Tumour node metastasis

### Independent prognostic factors in the training dataset

The univariate analysis demonstrated that age, sex, surgical treatment, grade, tumour size, T staging, N staging, M staging and TNM stage were statistically significant (Table 2). When we put these variables into the Cox PHs regression model, we found that age, sex, tumour size and TNM stage were independently correlated with prognosis. Table 3 shows the results of the variable selection with hazard ratios and *P*-values.

**Table 2:** Univariate analysis of the clinicopathological features of the training set

Variable	HR	***P-value***	95% CIs	
			Lower	Upper
**Age (years old)**				
≤ 50	1			
> 50	2.874	0.001	1.512	5.463
**Sex**				
Male	1			
Female	0.487	0.037	0.247	0.959
**Surgical treatment**				
Yes	1			
No	0.055	< 0.001	0.027	0.111
**Tumour size (cm)**				
< 1	1			
1–2	1.672	0.396	0.510	5.481
> 2	23.327	< 0.001	9.125	59.635
**G grade**				
G1	1			
G2	2.542	0.042	1.036	6.238
G3	25.403	< 0.001	13.082	49.327
**T staging**				
T1	1			
T2	3.303	< 0.001	1.221	8.937
T3	21.354	< 0.001	10.324	44.171
T4	26.040	< 0.001	10.007	67.764
**N staging**				
N0	1			
N1	10.053	< 0.001	5.582	18.105
**M staging**				
M0	1			
M1	9.701	< 0.001	5.389	17.464
**TNM stage**				
I	1			
II	2.199	0.237	0.595	8.130
III	15.105	< 0.001	6.527	34.956
IV	22.365	< 0.001	10.149	49.285

*HR* Hazard ratios, *CI* Confidence interval, *TNM* Tumour node metastasis

**Table 3:** Selected variables according to the Cox proportional hazards regression model

Variable	HR	***P-value***	95% CIs	
			Lower	Upper
**Age (years old)**				
≤ 50	1			
> 50	2.047	0.040	1.034	4.055
**Tumour size (cm)**				
< 1	1			
1–2	1.596	0.449	0.476	5.356
> 2	5.350	0.005	1.663	17.208
**G grade**				
G1	1			
G2	0.734	0.533	0.278	1.941
G3	4.154	0.001	1.770	9.750
**TNM stage**				
I	1			
II	0.592	0.471	0.142	2.460
III	2.771	0.054	0.983	7.807
IV	5.676	< 0.001	2.150	14.981

*HR* Hazard ratios, *CI* Confidence interval, *TNM* Tumour node metastasis

### Prognostic nomogram for OS

**Fig. 1:**
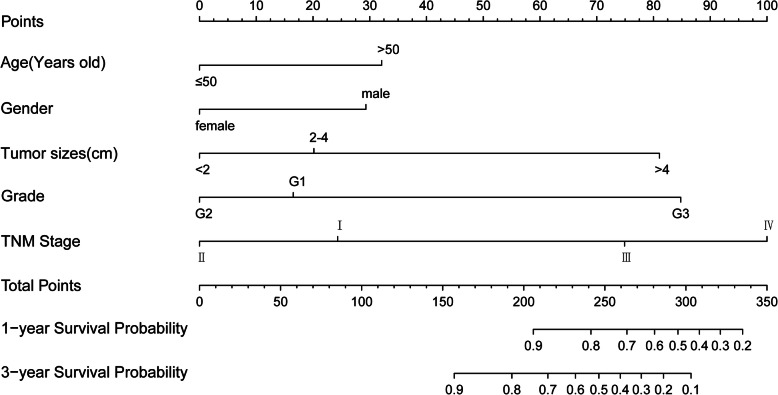
nomogram predicting 1- and 3-year OS of patients with rectal NETS. The nomogram sums the points identified on the scale for each variable. The total points projected on the bottom scale indicate the probability for 1- and 3-year overall survival

A nomogram was constructed based on the results of the Cox proportional hazards regression predicting 1- and 3-year overall survival (Fig. 1). Each point can be determined by drawing a line straight upward from each variable to the point axis. The total points are then calculated by summing each point to indicate the probability of 1- and 3-year survival.

### Comparison of predictive accuracy for OS between the nomogram and the TNM staging system or G grade system

The concordance index of the nomogram was 0.872 (95% CI, 0.806–0.938) for predicting the OS of the rectal NETS, which was superior to both predictions based on the seventh AJCC TNM classification and WHO grade guidelines, with concordance indices of 0.794 (95% CI, 0.721–0.866; *p* < 0.001) and 0.817 (95% CI, 0.752–0.881; *p* < 0.001), respectively. In the SEER validation sets, discrimination was also excellent. The C-index was 0.648 (95% CI, 0.611–0.684), which was superior to both predictions based on the seventh AJCC TNM classification and WHO grade guidelines, with concordance indices of 0.603 (95% CI, 0.571–0.635; *p* = 0.016) and 0.583 (95% CI, 0.547–0.619; *p* < 0.001), respectively.

### Comparison of the accuracy between the prediction by the nomogram and the actual observation for OS

**Fig. 2:**
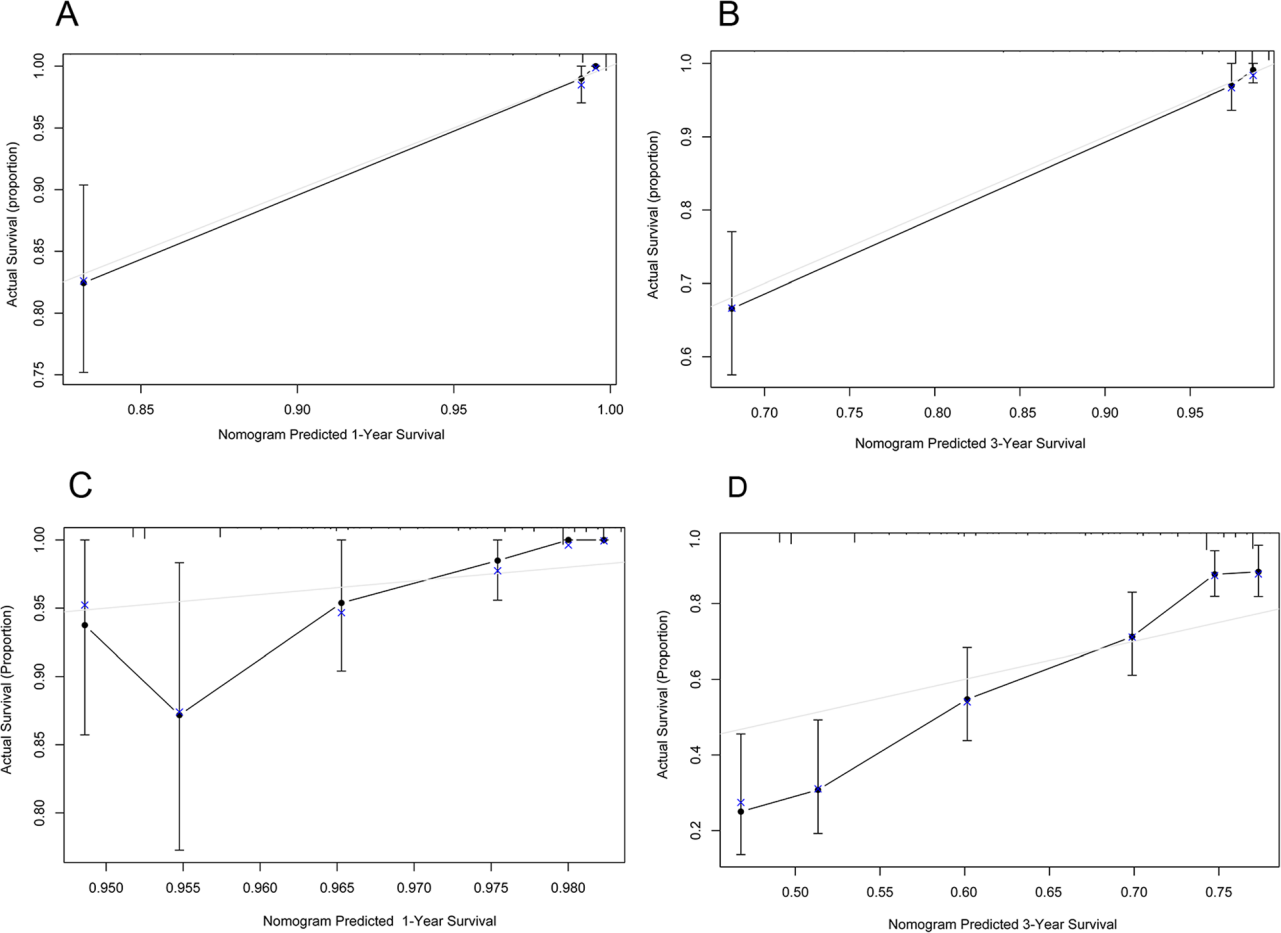
The calibration of the nomogram in the training and validation sets. The x-axis represents the survival rate predicted by the nomogram, whereas the y-axis presents the actual survival rate. The 95% CIs were measured via a Kaplan-Meier analysis. All predictions lie within a 10% margin of error. **a** 1-year OS in the training set. **b** 3-year OS in the training set. **c** 1-year OS in the validation set. **d** 3-year OS in the validation set

Figure 2 is a calibration diagram of nomogram. The x-axis represents the predicted survival rate calculated by nomogram, and the y-axis represents the actual survival rate estimated by Kaplan Meier. The graph shows that the actual survival rate is closely related to the predicted survival rate and is always within the error range of 10%.

## Discussion

Neuroendocrine tumour (NETs) is a relatively rare tumour, and the incidence of neuroendocrine tumours in the United States was 5.25/100000 [1] in 2004. According to the SEER database, the incidence of gastrointestinal NETs has been increasing in recent years [13]. The incidence of rectal NETS rates is the highest in the gastrointestinal tract, accounting for approximately 29% [3] of gastrointestinal NETs. However, most NETs seem to be sporadic, and risk factors for sporadic NETs are poorly understood.

There are few studies that focus on rectal NETs and these studies all contain limited patient cohorts [14–16]. Our study included 310 patients with rectal NETs and is the largest Chinese cohort so far.

With the application and popularization of endoscopic techniques, rectal neuroendocrine tumours are diagnosed earlier currently, and most of them are treated with endoscopic surgery. Of course, if the tumour size is large or there are metastatic lymph nodes, more extensive surgery is indicated. We can see that the tumour size or lymph node involvement will influence the surgical procedure and influence patient outcomes. However, there are several other clinicopathological features that can influence patients’ outcomes. According to published data and our analysis, grade, the depth of tumour invasion (T), the number of metastatic lymph nodes (N), distant metastases (M) and age at diagnosis are factors that influence outcomes. Chi et al. [17] found that tumour grade was an independent prognostic factor, while Weinstock et al. [14] found that tumour stage was an independent prognostic factor, and Chagpar et al. [18] found that the depth of tumour invasion, tumour size, lymph node metastasis and distant metastasis were independent prognostic factors.

When we discuss prognosis, all of the elements above should be taken into consideration. However, the most common predictive systems, namely, the TNM classification and grade, only focus on a portion of these variables and sometimes these two classifications conflict. For example, if a patient has a grade 1 tumour with liver metastasis, according to the grade predictive system, this patient is low-grade and has a good prognosis. In contrast, when we put this patient into the TNM system, it is a late-stage tumour and the patient has a poor prognosis. Clearly, these two systems are limited in predicting patient outcomes.

However, nomograms can take these variables into account in a Cox PHs regression. However, only a few nomogram studies have focused on NETs. Modlin et al. [19] focused on small-intestinal neuroendocrine tumours and Ye L et al. [20] built a nomogram to predict outcomes for pancreatic neuroendocrine tumours. However, these studies have relatively small samples and do not include rectal NETs. This study presents the first nomogram for predicting the survival of patients with rectal NETs.

This nomogram includes both grade and TNM stage, thereby addressing some of the limitations of the other predictive models. As expected, the predictive accuracy of the nomogram was superior to both the predictions of the TNM classification and the WHO grade guidelines, with concordance indices of 0.872 compared with 0.794 and 0.817, *p* < 0.001, respectively.

As for age and tumour size, we found that they were both important elements that influence prognosis. Zhang X et al. [21] reported that young age was a favourable prognostic factor, while Li P et al. [22] reported that lymph node metastasis was related to the tumour diameter and furthermore influenced the prognosis of rectal NETs. In our study, we found that patients likely had a decreased rate of survival with increasing tumour size.

It seems that Ki-67 or mitotic rate per 10 high-power fields could be better variables because they are continuous variables that have a wider range of values and can be more individual compared with the categorical variables. However, we combined these two variables as grade in order to simplify this model and to make sure this nomogram can be used easily.

This study has some limitations. One is that we did not include functional status or treatment as variables. According to the NCCN guidelines, patients with metastatic neuroendocrine tumours and carcinoid syndrome should be treated with somatostatin analogues [23]. However, even though our 5 hospitals are the largest medical centres in southern China, medical resources are limited. Some patients could not wait to receive continuous therapy and went to other hospitals for treatment. Others declined treatment secondary to cost or due to a lack of understanding. Given these limitations, we opted to not include these variables to not compromise the current form of the nomogram.

Another limitation was that most of the patients were diagnosed within the last 3 years as this disease has become more widely recognized. With the routine use of endoscopy, the incidence of rectal NETs has been increasing in recent years, but given the lack of patients with long-term follow-up, we could not include the 5-year overall survival rate. With time, we can collect more patients and variables and improve upon the nomogram.

## Conclusion

We have developed an individualized nomogram for precisely predicting OS for patients with rectal NETs. Its advantages as a prognostic tool when compared to traditional TNM staging systems or WHO grade classifications should allow it to make a significant clinical impact in the near future.

## Data Availability

The datasets used and analysed during the current study available from the corresponding author on reasonable request. You can go through the listed link and email the corresponding author for a password and get the data. https://pan.baidu.com/s/1gYw6xmg1swLSHV-nJduU8Q.

## Abbreviations

NETs: Neuroendocrine tumours
SEER: Surveillance, Epidemiology, and End Result
TNM: Tumour Node Metastasis
AJCC: American Joint Committee on Cancer
ENETS: European Neuroendocrine Tumour Society
NCCN: National Comprehensive Cancer Network
OS: Overall survival
